# Predation of Daurian redstarts offspring in nest boxes by the Oriental magpie‐robin and tree sparrow

**DOI:** 10.1002/ece3.10093

**Published:** 2023-05-11

**Authors:** Guixia Wan, Huahua Zhao, Xizhu Liu, Longwu Wang, Wei Liang

**Affiliations:** ^1^ School of Life Sciences Guizhou Normal University Guiyang China; ^2^ Ministry of Education Key Laboratory for Ecology of Tropical Islands, Key Laboratory of Tropical Animal and Plant Ecology of Hainan Province, College of Life Sciences Hainan Normal University Haikou China

**Keywords:** cavity‐nesting birds, *Copsychus saularis*, nest box, nest predation, *Passer montanus*, *Phoenicurus auroreus*

## Abstract

Birds select suitable nest sites for breeding to ensure their own and offspring's survival; however, they inevitably suffer some potential predation risk. We studied the breeding ecology of Daurian redstarts (*Phoenicurus auroreus*) by providing nest boxes for their breeding from March to August of 2022. We recorded the predation of both Daurian redstarts eggs or nestlings by Oriental magpie‐robins (*Copsychus saularis*) and tree sparrow (*Passer montanus*). Oriental magpie‐robin were recorded attacking a feeding female adult and depredating nestlings. After the nestling predation event, the Daurian redstarts abandoned the nest. This video evidence provide a better understanding of the potential predators of cavity‐nesting birds.

## Graphical abstract

The preying on the egg or nestling of Daurian redstarts by Oriental magpie‐robins (*Copsychus saularis*) and tree sparrow (*Passer montanus*) was recorded, of which the Oriental magpie‐robin attacking a feeding female adult and depredating the nestling of the Daurian redstarts.

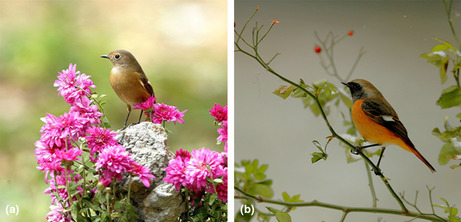

## 1 INTRODUCTION

**FIGURE 1:**
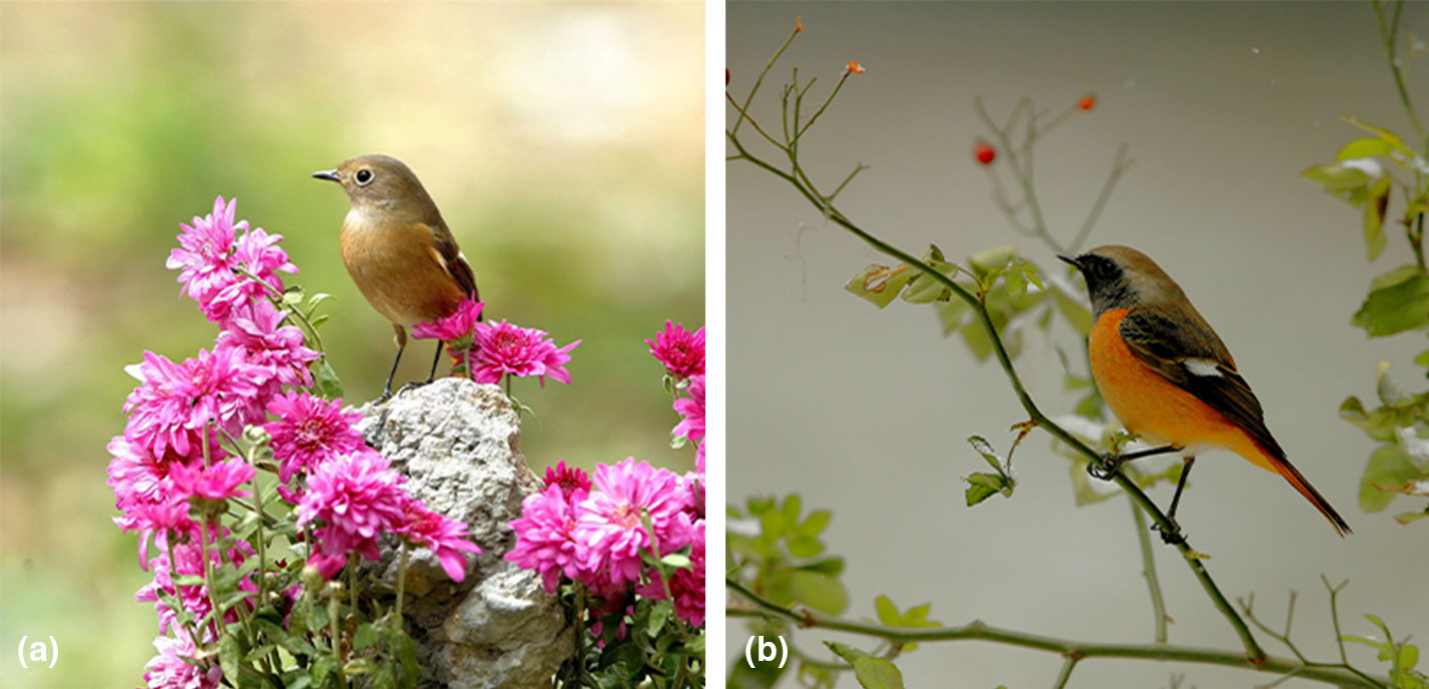
Records of Daurian redstart in this study (a, female; b, male; photos from Jian Yang).

Predation is one of the most important selective pressures in nature, shaping evolutionary relationships in many systems including birds (Caro, 2005). The life history of all birds is characterized by a critical stage in which they are bound to a nest during the breeding period. The selective pressures acting during this period probably modulate their biology to a large extent; thus, it is not surprising that nest predation is considered a key source of selection for birds (Martin, 1995). Although the concealment and of cavity‐nesting providing protection for cavity‐nesting birds, nest predation remains a major cause of failure in their breeding process (Lima, 2009; Martin, 1993; Martin & Li, 1992). The species and kinds of nest predator depend on the geographical areas and habitat types (Shen et al., 2023). Therefore, the mode of predation and selective forces’ pressures on nest sites may vary (Czeszczewik, 2004; Picman & Schriml, 1994). For example, some snakes can accurately swallow birds' eggs or nestlings in the nest (Gartner & Greene, 2008), and some raptors may prefer to attack the nestlings from outside the nest entrance using their beaks or claws when they catch the nestlings (Barnett et al., 2013; Suzuki & Ueda, 2013). Due to the constraint size of the cavity entrance, the predators of cavity‐nesting birds mainly include some snakes, rodents, mustelids (Wesolowski, 2002); however, cavity‐nesting birds as predators are rare, with some woodpeckers being reported in field (Wesolowski, 2002). Knowing the identity of predators and their mode of predation could help us to predict the prevalence of nest loss (Cox et al., 2012) and better understand ecological interactions and establish their conservation goals (Chalfoun et al., 2002; Lima, 2002). It also contributes to our understanding of the selection pressures that influence parental and offspring anti‐predator strategies (Ibáñez‐Álamo et al., 2015). However, the frequency and discreetness of natural predation events make it difficult for us to witness the predation process directly. Researchers often speculate about potential predators based on the remains (Williams & Wood, 2002), which may lead to the consequence that the actual nest predators are different from the predicted predators (Peterson et al., 2004). Miniature video cameras are widely used to monitor the behavior of breeding birds. For example, the video recording showed the process of multiple host individuals of the Oriental reed warbler (*Acrocephalus orientalis*) mobbing and at tacking a female common cuckoo (*Cuculus canorus*) in the field (Wang et al., 2020, 2023; Zhao et al., 2022). In addition, the video cameras are able to accurately capture the process of nest predation cases (Ball & Bayne, 2012). In this study, we recorded the whole process of Oriental magpie‐robins (*Copsychus saularis*) (hereafter OMR) and tree sparrow (*Passer montanus*) (hereafter TS) preying on the egg or nestling of Daurian redstarts (*Phoenicurus auroreus*) (hereafter DR) in next boxes. Here, we report this information in detail. The DR belongs to the order Passeriformes, family Turdidae, and is distributed in all provinces of China except Xinjiang, Tibet, and Qinghai. Its clutch size is usually 3–5, both male and female parents were involved throughout the brood (Figure 1). The DR usually nest in door joints, house pillars, road stone joints, and house wall joints, among other locations.

## 2 MATERIALS AND METHODS

The study site was located in Liuzhi (26°10′–14′ N, 105°13′–24′ E, 1500 m in elevation), Liupanshui City, Guizhou Province, southwestern China. We studied the breeding ecology of DR by hanging nest boxes for them on trees or electric poles 3–4 m above the ground from March to August 2022. The entrance hole size of artificial nest boxes was 14 × 11 cm. The nest boxes have these dimensions: The height is 26 cm, on 12 × 12 cm. We used a miniature video camera (Uniscom‐T71, 70 × 26 × 12 mm; Mymahdi Technology Co. Ltd.) to record the behavior of breeding birds during egg incubation and brood rearing. The miniature camera was mounted at the top, inside the nest box, and a mobile power supply (20,000 mAh, Romoss‐Sense 6; Romoss Technology Co. Ltd.) was used to ensure all‐day recording. After the nest boxes were hung, they were all checked every 5 days. Once a DR was found in a nest box, we numbered the nests and recorded the parameters of egg production and brood rearing.

## 3 RESULTS

In total, 143 nest boxes were suspended, of which 48 (36.5%) were used by breeding birds. Specifically, 28 (19.6%) were occupied by the OMR, 9 (6.3%) by the DR, 11 (7.7%) by the TS, and four (2.8%) by the Japanese Tit (*Parus minor*). Through video analysis, four DR nests were recorded as being predated by other cavity‐nesting birds, of which three nests were predated by the OMR and one nest was predated by the TS.

**FIGURE 2:**
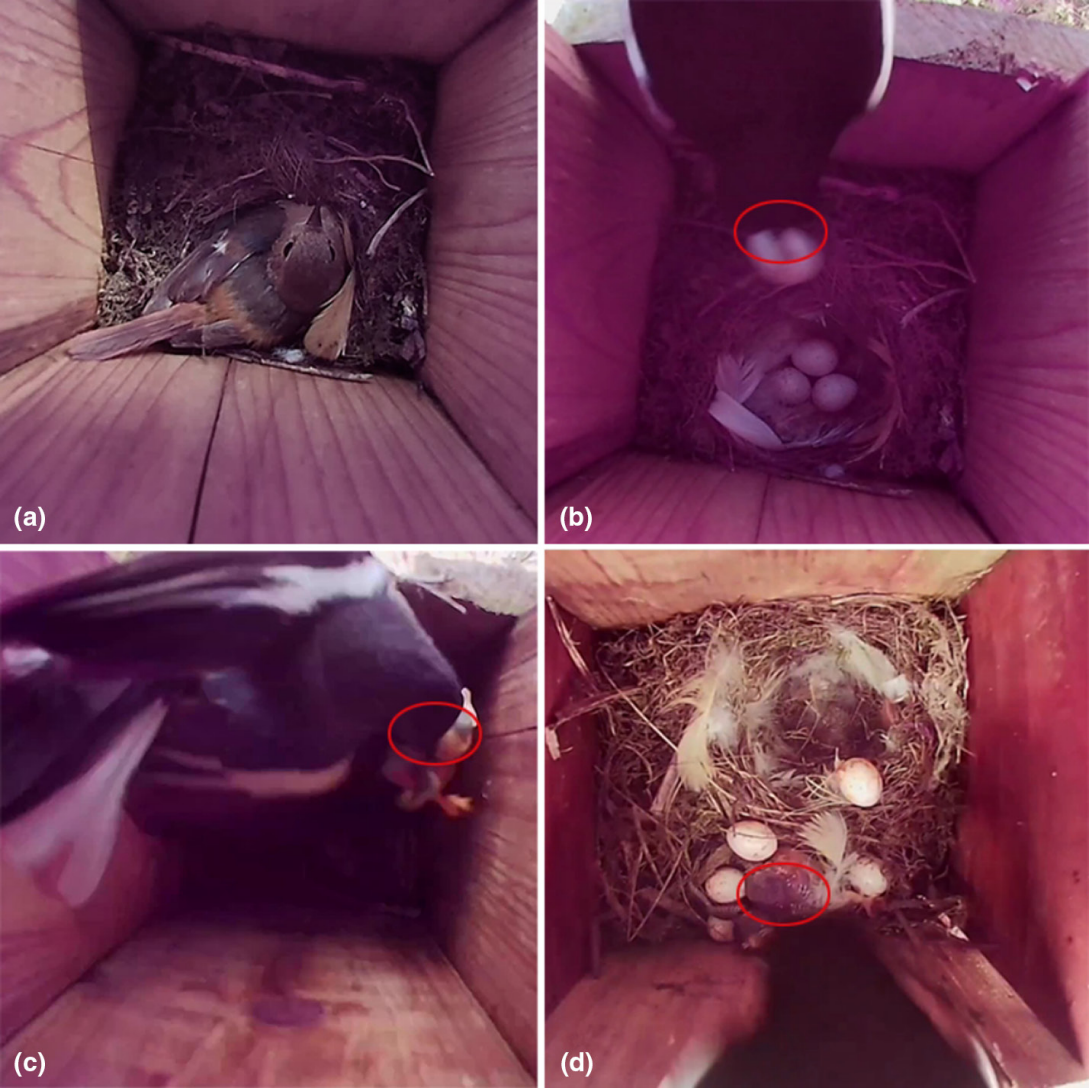
Diagram of the Oriental magpie‐robin preying on the eggs or nestlings of Daurian redstarts (a, the female Daurian redstart incubating eggs; b, the Oriental magpie‐robin preyed on one egg out of the nest box (details in Video S1); the red circle refers to the egg; c, the Oriental magpie‐robin preyed on the 5‐day‐old Daurian redstart nestling from the nest box (details in Video S2); the red circle refers to the nestling; d, the Oriental magpie‐robin entered the nest box and used its beak to peck the head of the common 3‐day‐old nestling of the cuckoo (details in Video S3); the red circle refers to the nestling).

**FIGURE 3:**
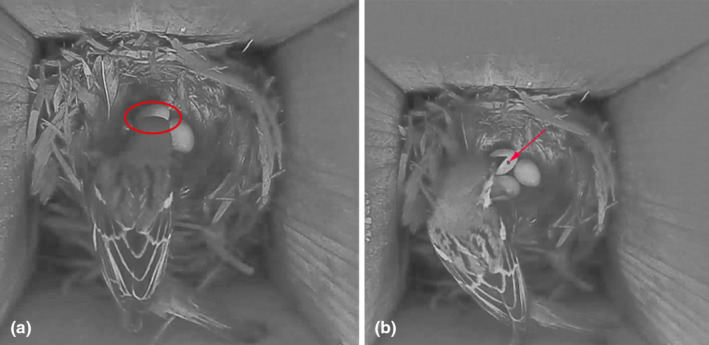
The tree sparrow preying on the egg of the Daurian redstart (a, the tree sparrow entered the nest box and used its beak to peck the egg; the red circle refers to the egg; b, one poked egg in the nest that was pecked by the tree sparrow (details in Video S4); the red arrow refers to the poked egg).

At 9:59 h on July 4 (local time, Beijing, GMT+8), an OMR preyed on a DR egg, and the entire process took 7 s in R1 (Table 1; Figure 2b, and see Video S1). This nest did not give up because of the loss of the egg. At 16:53 h on June 26, an OMR preyed on a 5‐day‐old DR nestling in R2 (Table 1; Figure 2c, and see Video S2). Finally, another nestling was found dead in the nest. We confirmed that the DR had abandoned the nest and suspended video monitoring of this nest box. At 14:12 h on June 26, an OMR stabbed a 3‐day‐old common cuckoo (*Cuculus canorus*) nestling in R3, which parasitized the nest of a DR, in the head with its bill. The OMR continuously stabbed the nestling until it had died while also stabbing a DR egg. At 14:14 h, it preyed on this nestling (Table 1; Figure 2d, and see Video S3). At 16:50 h, a male DR brought food back to the nest and observed the destroyed nest for a period of time. At 17:50, a female DR returned again to observe and confirm the nest condition, and the parent birds never returned thereafter. At 13:15 h on July 7, a TS entered a DR nest and then stabbed the egg with its beak in R4 (Figure 3a). The entire process took 65 s. The TS confirmed that the DR eggs were stabbed and then left (Table 1; Figure 3b, and see Video S4). This nest did not give up after destroying the egg.

**TABLE 1:** The Oriental magpie‐robin and tree sparrow preying on the offspring of the Daurian redstart.

Nest ID	Clutch size	Intruder species	Intruder behavior	Date	Video evidence
R1	Four eggs	Oriental magpie‐robin	Prey on egg	July 4, 2022	Video S1
R2	Three nestlings	Oriental magpie‐robin	Prey on nestling	June 26, 2022	Video S2
R3	Five eggs + one nestling	Oriental magpie‐robin	Kill + prey on *Cuculus canorus* nestling	June 26, 2022	Video S3
R4	Three eggs	Tree sparrow	Prey on egg	July 7, 2022	Video S4
R2	Three nestlings	Oriental magpie‐robin	Attack Daurian redstart	June 26, 2022	Video S5

In the face of intruders, parent DR birds showed nest defense behaviors. At 16:13 h on June 26, an OMR tried to invade the nest of a DR in R2. Coincidentally, a female Daurian redstart was feeding; thus, the DR and the OMR fought directly, and at the same time, a strong and noisy alarm sounded (detail in Video S5).

## 4 DISCUSSION

We incidentally observed that the OMR and the TS preyed on DR eggs or nestlings, of which one OMR attacked feeding DR and successfully preyed on DR nestlings in this study. All predations of DR nests were recorded on videos, adding to the compelling evidence of actual cases of nest predation in secondary cavity‐nesting birds.

Japanese tit (*Parus minor*) has evolved a variety of nest defense behaviors to prevent their offspring and themselves from being harmed by predators, such as alarm calls, mobbing, and attacks (Yu et al., 2021). Similarly, DR showed alarm calls and nest defense behaviors to prevent their offspring and themselves from being harmed by predators (unpubl. data from our field observations). For many marsh‐nesting blackbirds, predation is considered to be an important source of egg and nestling mortality (Ricklefs, 1969), the Yellow‐headed Blackbirds (*Xanthocephalus xanthocephalus*) has the similar encounter (Westneat, 1992). Predators can be responsible for most of brood losses and breeding losses could reach even the level of two‐thirds of breeding attempts among cavity nesters (Walankiewicz, 2002). Our results indicate that the OMR is an important predator of DR in this study area and an important cause of DR loss of offspring, and apparently, they are responsible for DR nest mortality. TS may attack DR nests purely to predate eggs, or alternatively attacking the nest might also be beneficial through relieving the pressure of inter‐species nest competition. In conclusion, it was the first observation on an OMR attacking a feeding female adult and depredating the nestling of the DR in a nest box. In addition, this observation gives us a better understanding of the potential predators of the cavity‐nesting birds. Our observation confirms that continuous video monitoring of nests is reliable and practical method to record predators and anti‐predation behaviors (Benson et al., 2010; Weidinger, 2009).

## AUTHOR CONTRIBUTIONS

**Guixia Wan:** Data curation (equal); formal analysis (equal); writing – original draft (equal). **Huahua Zhao:** Data curation (equal); methodology (equal). **Xizhu Liu:** Data curation (equal); investigation (equal). **Longwu Wang:** Supervision (equal); writing – review and editing (equal). **Wei Liang:** Supervision (equal); writing – review and editing (equal).

## FUNDING INFORMATION

This work was supported by the National Natural Science Foundation of China (Nos. 31960105, 32260253 to LW, 31970427, 32270526 to WL). LW was funded by the Guizhou Natural Science Foundation (No. ZK [2022]‐316), and WL supported by the specific research fund of The Innovation Platform for Academicians of Hainan Province.

## CONFLICT OF INTEREST STATEMENT

The authors declare that they have no competing interests.

## Supporting information


Video S1.

Video S2.

Video S3.

Video S4.

Video S5.

Video Captions

## Data Availability

Videos of two species of birds prey on the DR by interspecific killing behavior, and data in this manuscript are available at the Dryad Digital Repository: https://datadryad.org/stash/share/UHaV2MxRu8cEtBcUG8eaIOFXbQYkdi7DA5fyXn‐ULyQ.
