# Supplementary material for: Predation of Daurian redstarts offspring in nest boxes by the Oriental magpie‐robin and tree sparrow
Source: Ecol Evol. 2023 May 11;13(5):e10093. doi: 10.1002/ece3.10093 (PMC10172884; doi:10.1002/ece3.10093)
Supplement: Supplementary file 6 — Video Captions [file ECE3-13-e10093-s004.docx]

**Video** **S1.** The process of the Oriental Magpie Robin preying on one Daurian Redstart egg.

**Video** **S2.** The process of the Oriental Magpie Robin preying on one Daurian Redstart nestling at 5 days.

**Video** **S3.** The process of the Oriental Magpie Robin killing and preying on one 3-day-old cuckoo nestling.

**Video** **S4.** The process of the Oriental Magpie Robin preying on one Daurian Redstart egg.

**Video** **S5.** The process of the Oriental Magpie Robin attacking the female Daurian Redstart.
